# Does the femoral component design modified in consideration of Asian anatomical characteristics fit better than the conventional design for Korean femora in reality?

**DOI:** 10.1007/s00402-024-05593-y

**Published:** 2024-09-29

**Authors:** Seok Jin Jung, Han Sol Kim, Seung Joon Rhee, Sang Min Lee, Darryl D. D’lima

**Affiliations:** 1 Department of Orthopedic Surgery, Biomedical Research Institute, Pusan National University Hospital, 179 Gudeok-ro, Seo-gu, Busan, 602-739 Korea; 2 College of Medicine, Pusan National University, 179 Gudeok-ro, Seo-gu, Busan, 602-739 Korea; 3 Department of Orthopedic Surgery, Research Institute for Convergence of Biomedical Science and Technology, Pusan National University Yangsan Hospital, Yangsan, Korea; 4 Department of Molecular Medicine, Scripps Research, La Jolla, San Diego, CA 92037 USA; 5 Shiley Center for Orthopaedic Research and Education at Scripps Clinic, Scripps Health, La Jolla, San Diego, CA USA

**Keywords:** Knee, Replacement, Arthroplasty, Femur, Prosthesis, Asian patients

## Abstract

**Introduction:**

We aimed to investigate the anatomical and clinical advantages of an Asian-specific femoral component design with a high femoral aspect ratio, compared with the conventional femoral component design.

**Materials and methods:**

A retrospective analysis of the operation and outpatient clinic records of 239 knees operated on using an anatomically modified femoral component design (MFCD, Group A) and 153 knees operated on using a conventional femoral component design (CFCD, Group B) in Korean patients was performed. Three subgroups were created based on the mediolateral size of the two different femoral component designs. The geometric accommodation of each femoral component was assessed using intraoperatively measured femoral posterior condylar resection and posterior condylar trimming amounts. Clinical outcomes were assessed using a range of motion (ROM) and patient-reported outcome measurements.

**Results:**

In the comparison between Groups A and B, the mean combined bilateral posterior condylar trimming (XPCT) was 2.91 [2SD: − 4.12–9.94] and 1.45 [2SD: − 5.89–8.80], and the median XPCT was 3 and 1.5. In the largest subgroup (subgroup 2), Groups A and B included 100 and 112 patients, all six posterior condylar resection and trimming parameters were significantly larger in Group A. Preoperative and postoperative ROM and Hospital for Special Surgery scores were similar between the two groups. Preoperative Western Ontario and McMaster Universities Arthritis Index (WOMAC) was higher in Group A. However, postoperative WOMAC was similar between the groups. Perioperative improvement in WOMAC index was significantly greater in Group B.

**Conclusions:**

The Asian-specific femoral component design resulted in more resection and trimming of the femoral posterior condyle than the conventional design despite it was not associated with different clinical outcomes. Surgeons should be aware of unexpected excessive posterior condylar resection and formation of large flexion gap when using femoral component design with high femoral aspect ratio.

## Introduction

Total knee arthroplasty (TKA) is a globally performed surgical treatment of end-stage knee osteoarthritis (OA). In the US, over 100,000 annual procedures are performed annually, and the number of cases is gradually increasing. The trend of incrasing TKA in Korea is concordant with US, and around 50,000 cases are performed annually. Accordingly, TKA implant designs have constantly improved over the decades [1–4].

Among the numerous design modifications in TKA implants, some features accommodate anthropometric characteristics that differ between sexes or races. Gender-specific implant designs were developed during the mid-2000s based on the literature highlighting sex differences in the distal femoral anatomy, namely narrower mediolateral (ML) to anteroposterior (AP) aspect ratio and less pronounced anterior femoral condyles [5–8]. The next anthropometric consideration in the design modification was a race-specific design to accommodate anatomical differences between Caucasian and East Asian populations [7, 9–15]. Implant designs reflecting characteristic anatomical features of the distal femur in Asians with higher mediolateral-to-anteroposterior (ML/AP) ratios have been developed and are currently available in the market [16–18].

Selecting femoral component size in TKA requires consideration of differences in the preoperative and intraoperative references of femoral sizing. Preoperative measurements are primarily based on plain radiography, and the AP diameter of the distal femoral condyle in the sagittal plane is mainly used to determine femoral component size [19]. In contrast, the ML diameter of the distal femur or anterior flange is hardly recognizable due to the overlapping structures. However, intraoperatively, the ML diameter of the distal femoral resection surface and anterior flange dictates femoral component size among the preoperatively determined size selections [2, 16, 20]. Hence, femoral component sizing is subject to several variables, and predicting the influence of different femoral aspect ratios on femoral component designs is challenging.

There is a paucity of literature proving whether an Asian-specific modified femoral component design is appropriate for the shape of the Asian femora in practice. In this study, we aimed to analyze whether the modified femoral component design fits better than the conventional design for Asian femora from a geometrical perspective, and to evaluate the clinical outcomes compared with the conventional design.

Our first hypothesis was that the femoral implant with Asian specific modified femoral component design would replace femoral bilateral posterior condyles better with less over- or under-resection of the host bone compared to conventional femoral implant design of similar mediolateral dimension in Korean femora. The second hypothesis was that the choice of narrow version implant would be lower in modified femoral component design than in conventional femoral implant design if the basic femoral aspect ratio of the femoral component was fitter in Korean population. Thirdly, we hypothesized that Korean patients who were operated using modified femoral component design will show better clinical outcome in terms of knee range of motion and patient-reported outcome scores.

## Materials and method

**Fig. 1:**
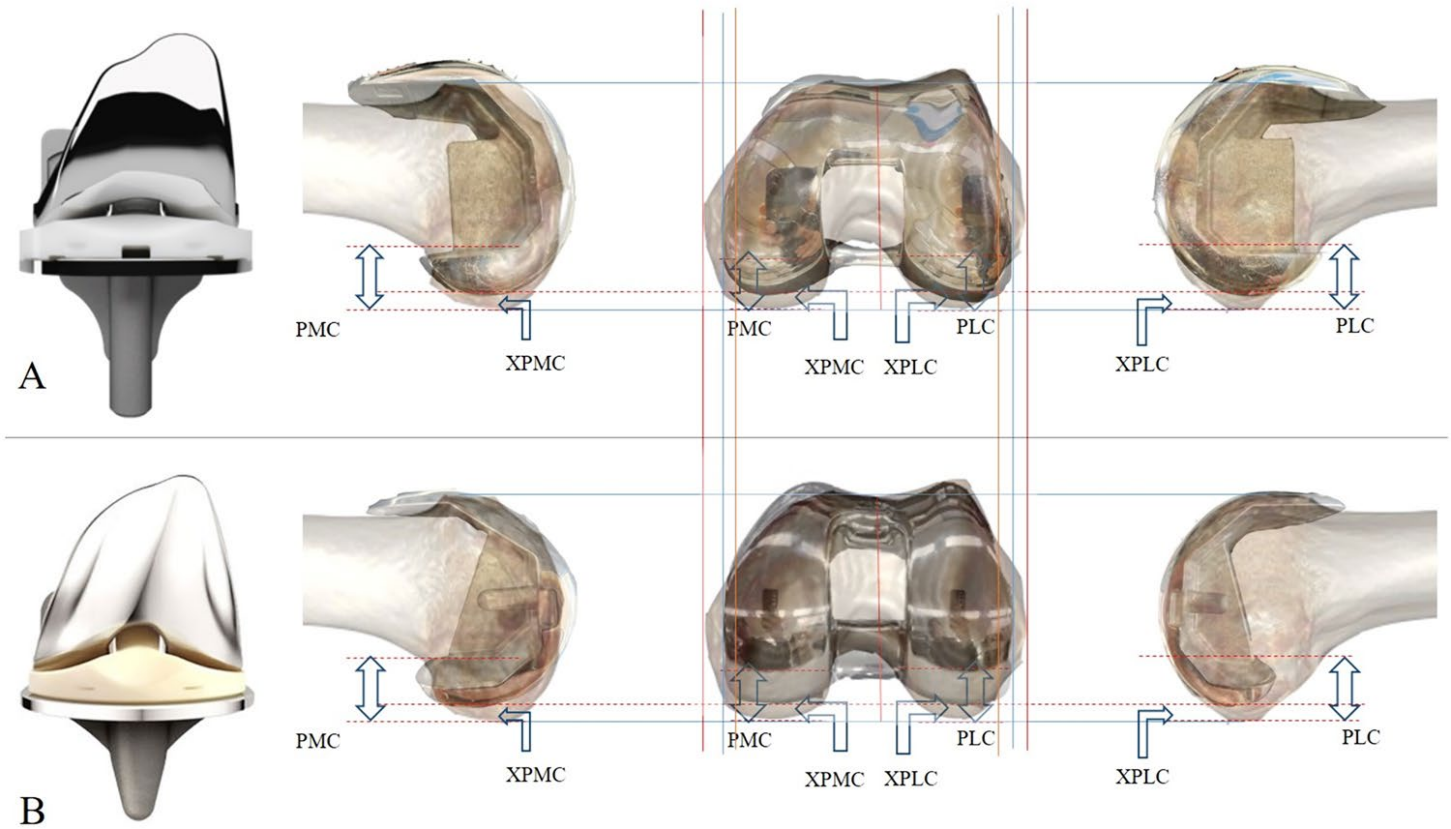
Posterolateral condylar resection (PLC), posterolateral condylar trimming beyond the implant posterolateral condylar thickness (XPLC), posteromedial condylar resection (PMC), and posteromedial condylar trimming beyond the implant posteromedial condylar thickness (XPMC) in Groups A (**A**) and Group B (**B**)

We retrospectively reviewed the surgical records of 828 knees in 701 Korean patients who underwent TKA at our institution between 2016 and 2022. Among them, 239 knees were operated on using an Asian-specific modified femoral component design (Group A: Anthem^®^ Total Knee System, Smith & Nephew, Memphis, TN, USA), and 153 knees were operated on using a conventional femoral component design (Group B: Attune^®^ Knee System, Depuy-Synthes, Warsaw, IN, USA). 369 knees operated using other TKA designs and 67 knees with posttraumatic or congenital deformities or bone destruction due to subchondral osteonecrosis were excluded. Patients with diagnosis of osteoarthritis and rheumatoid arthritis were enrolled for this study. There were 42 grade 2, 171 grade 3, and 179 grade 4 by Kellgren-Lawrence grading system in the enrolled patients. An orthopedic surgeon with over 10 years of knee surgery and arthroplasty experience performed all surgeries. The operative procedure followed a modified measured resection policy, using posterior cruciate ligament substituting type implant in both groups. Distal femoral resection was performed independently using an intramedullary guide, followed by proximal tibial resection using an extramedullary guide. Anteroposterior femoral bone resection was performed using the anterior referencing technique, considering the flexion gap. The anterior reference point was the most prominent edge on the anterolateral condylar ridge, smoothly connecting to the anterior cortex of the distal femoral shaft segment. The degree of femoral component external rotation was basically determined between the patient’s anatomical and surgical transepicondylar axis (TEA) to better accommodate the desired flexion gap morphology. The selection of the femoral component size was based on the integration of the femoral sizing guide indication, considering the flexion gap and avoiding implant overhang among ten available sizes in both groups. Regarding the mediolateral overhang, we attempted to avoid implant overhang using the largest covering size possible for all cases. The extent of posterior condylar bone resection was measured using a manual caliper (Townley Femur Caliper, IntegraJarit Instruments, Plainsboro, NJ, USA) with an accuracy of 0.5 mm on the back table during surgery. The resected bone thickness was measured in the posterior medial condyle (PMC) and posterior lateral condyle (PLC) (Fig. 1). The documented thickness of the resected posterior condyle was compared between Groups A and B in the subgroups of compatible femoral component sizes with similar mediolateral widths. We regarded that less difference in combined posterior condylar resection amount relative to the replacing femoral component posterior condyles in similar ML sizes meant fitter anatomical geometry. We set the threshold of a difference under 2 mm in the ML dimension as the compatibility of the femoral component size in each group (A and B) to belong to the same subgroup. The 2 mm threshold was set because a maximum 1 mm difference in each medial and lateral side would indicate clinical compatibility in the same distal femoral dimension. Additionally, thickness of the combined posterior condylar resection in total (PCT), trimming amount of the host posterior medial condylar bone beyond posterior medial condylar thickness of the implant (XPMC), posterior lateral condylar trimming amount beyond posterior lateral condylar thickness of the implant (XPLC), and combined bilateral posterior condylar trimming amount beyond combined bilateral posterior condylar thickness of the implant (XPCT) were calculated and compared (Fig. 1). The subtraction to calculate XPLC and XPMC from PLC and PMC was 9.5 mm for each in Group A and 9 mm for each in Group B. We assessed knee range of motion (ROM) and patient reported outcome measures (PROMs) because the amount of femoral posterior condylar resection is directly related to the formation of a flexion gap in the surgical procedure of anteriorly referenced TKA, and the flexion gap contributes to postoperative ROM, which greatly influences patient-reported clinical outcomes [21]. Pre-operative and postoperative ROM using goniometer, Hospital for Special Surgery Score (HSS), and Western Ontario McMaster University Osteoarthritis Index (WOMAC) were assessed to compare clinical fitness of the different implant designs [22, 23].

This study was approved by the Institutional Review Board of our hospital (2309–011-131). Informed consent was obtained from all patients.

For statistical analysis, categorical variables were compared using Pearson’s χ^2^ or Fisher’s exact tests. Continuous variables were compared as the means ± standard deviations or medians (interquartile range) using 2-sample Student’s t-tests or the Mann–Whitney U tests. All statistical analyses were performed using the SPSS software. (version 27.0; IBM Corp., Armonk, New York, USA).

## Results

**Fig. 2:**
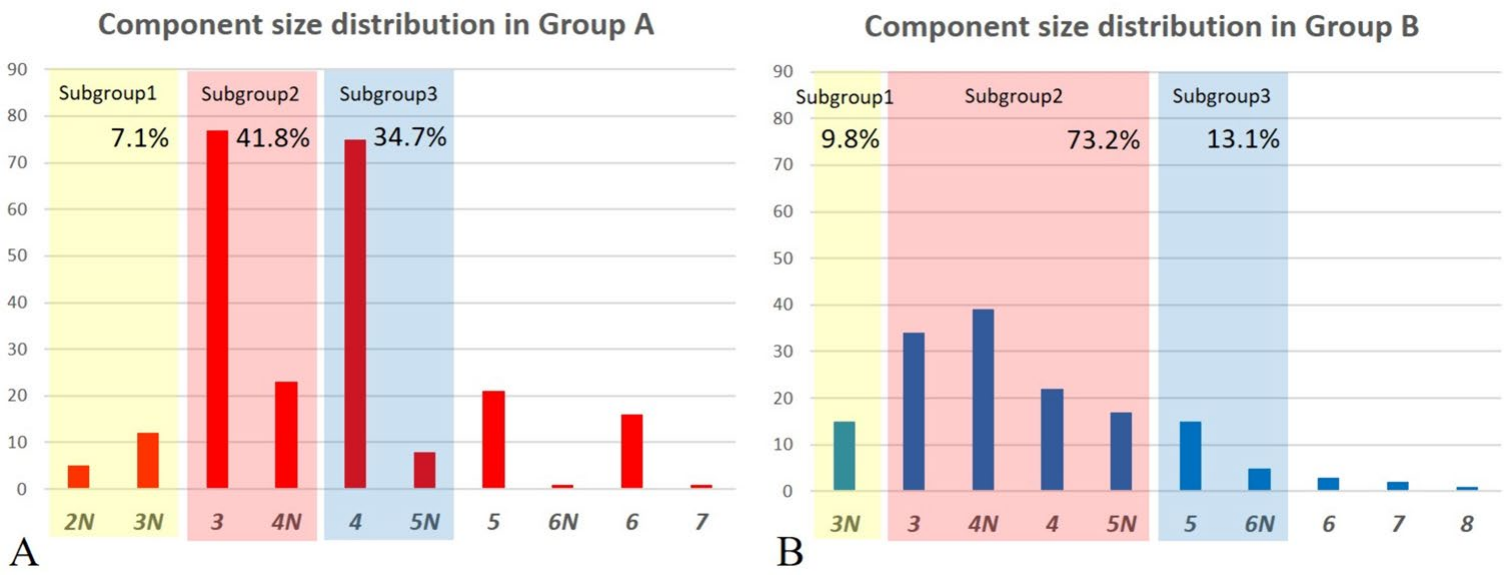
Number of knees in each femoral component size and ratio of each subgroup in the Group A (**A**) and Group B (**B**)

**Fig. 3:**
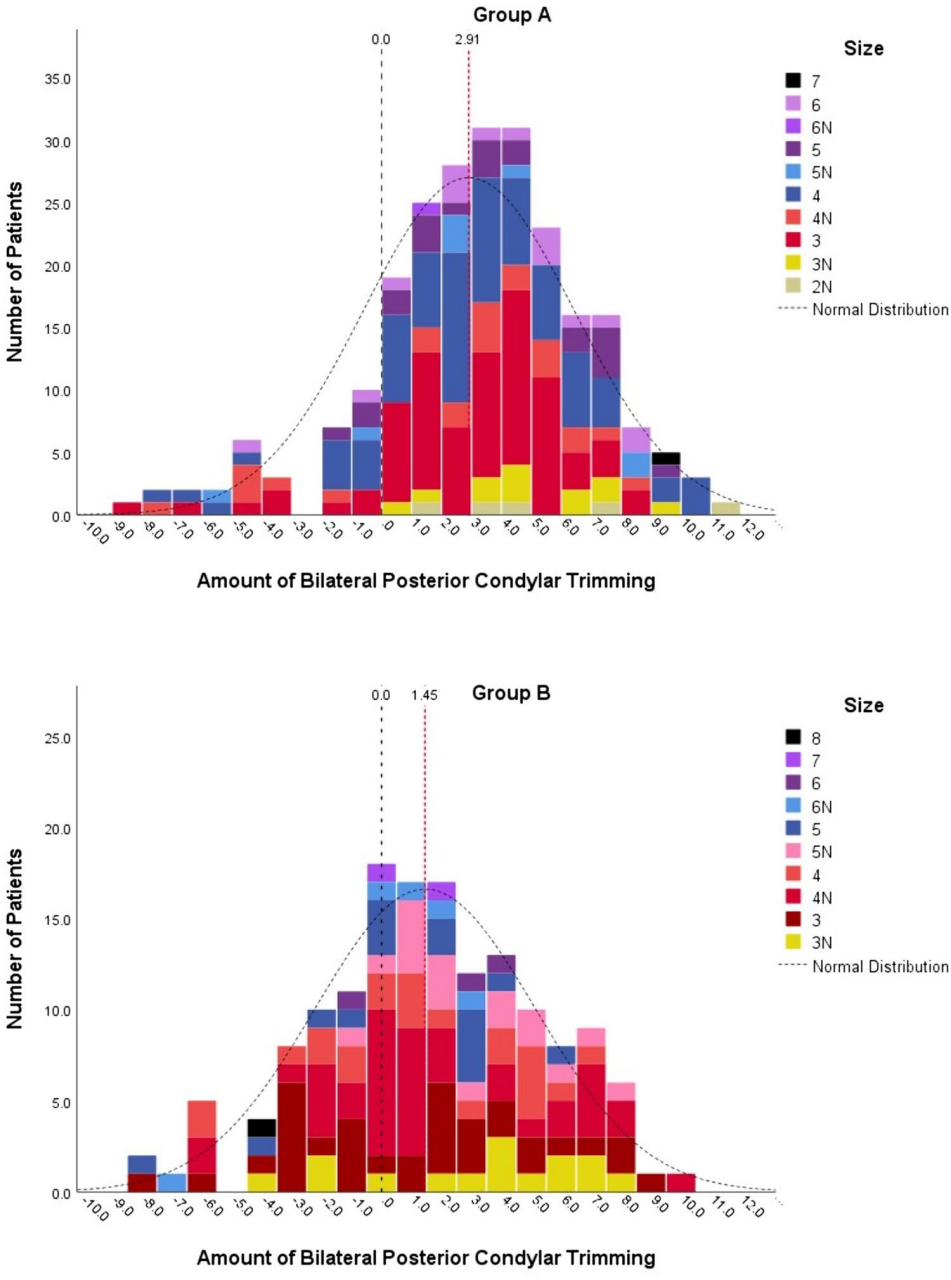
Distribution of bilateral posterior condylar trimming beyond the implant bilateral posterior condylar thickness (XPCT) according to each femoral component size in the Group A (**A**) and Group B (**B**)

Of the 239 knees in Group A, 41 were men, and 198 were women. The mean age was 71.8 years (range, 51–87 years), and the mean hip-knee-ankle axis (HKA) was 8.86° (range, − 15.2°–30.9°). Group A comprised 121 left and 119 right knees. Of the 153 knees in Group B, 21 were men, and 132 were women. The mean age was 69.6 years (range, 57–89 years), and the mean HKA was 7.68° (range, − 23.2°–24.6°). There were 77 left and 76 right knees in Group B. All demographic values, except for age (*p* = *0.001*) and BMI (*p* = *0.038*), were not statistically different between Groups A and B (Table 1). The number of knees in each femoral component size in group A from size 2 narrow (*2N*) to size *7* were 5(*2N*), 12(*3N*), 77(*3*), 23(*4N*), 75(*4*), 8(*5N*), 21(*5*), 1(*6N*), 16(*6*), and 1(*7*). In Group B from size *3N* to size *8*, the number of knees were 15(*3N*), 34(*3*), 39(*4N*), 22(*4*), 17(*5N*), 15(*5*), 5(*6N*), 3(*6*), 2(*7*), and 1(*8*). Among them, compatible ML sizes for the two different femoral component designs were identified (Tables 2 and 3) (Fig. 2). Although six subgroups were created according to the numerical specifications of the two different femoral component designs, analyses were only performed upon the three largest subgroups. Subgroup one included femoral components with an ML width between 56.0 and 58.0 mm. In subgroup one, Group A included 17 patients, and Group B included 15. PLC and PCT significantly differed as PLC 10.50 ± 1.39 and 8.83 ± 1.84 (*p* = *0.020*), PCT 23.79 ± 2.97 and 21.03 ± 3.61 (*p* = *0.027*) in Groups A and B, respectively. However, the remaining condylar resection parameters were similar between the groups in subgroup one. Subgroup two, which had the largest population, included femoral components with an ML width between 59.9 and 64.0 mm, and Groups A and B included 100 and 112 patients, respectively. PLC, PMC, PCT, XPLC, XPMC, and XPCT was significantly larger in Group A than Group B. Subgroup three included 83 patients in Group A and 20 in Group B, and comprised of femoral components with an ML width between 66.0 and 67.0 mm. Also in subgroup three, all six posterior condylar resection parameters in Group A was significantly larger than Group B (Table 4). Approximately 1 mm more trimming for the unilateral posterior condyle was observed if we considered the mean difference in XPCT between Groups A and B as 1.76, 2.01, and 2.32 in subgroups one, two, and three, respectively. In the comparison between entire Groups A and B, the mean XPCT was 2.91 [2SD: − 4.12–9.94] and 1.45 [2SD: − 5.89–8.80], and the median XPCT was 3 and 1.5, respectively (Fig. 3A, B). XPCT significantly differed in the sex-differentiated comparison between Groups A and B. Comparison between the sexes showed no difference in Group A. In contrast, XPCT in women was significantly larger than that in men in Group B (Table 5).

**Table 1:** Patient demographics and Preoperative parameters

Parameter	Group A (n = 239)	Group B (n = 153)	*p*-value
Sex			*0.264*
Women	198	132	
Men	41	21	
Age	71.8 ± 6.6 (51 ~ 87)	69.6 ± 6.3 (57 ~ 89)	0.001*
Side			*0.919*
Left	121	77	
Right	118	76	
Diagnosis			*0.447*
OA	197	130	
RA	42	23	
Kellgren-Lawrence grade			*0.372*
2	20	22	
3	103	68	
4	116	63	
BMI	26.2 ± 3.3 (18.4 ~ 37.3)	25.5 ± 3.4 (19.6 ~ 44.0)	0.038*
BMD	− 1.49 ± 1.04 (− 4.10 ~ 1.40)	− 1.56 ± 1.24 (− 4.00 ~ 2.30)	*0.581*
HKA	8.86 ± 6.68 (− 15.20 ~ 30.80)	7.68 ± 7.64 (− 23.20 ~ 24.60)	*0.131*
ROM	115.5 ± 16.6 (50.0 ~ 135.0)	114.8 ± 19.0 (25.0 ~ 135.0)	*0.729*
HSS	62.9 ± 12.8 (30.0 ~ 91.0)	61.2 ± 12.7 (8.0 ~ 93.0)	*0.219*
WOMAC	52.1 ± 17.8 (7.0 ~ 92.0)	57.8 ± 17.3 (14.0 ~ 96.0)	0.003*

*BMI* body mass index, *BMD* bone mineral density, *HKA* hip-knee-ankle axis, *ROM* range of motion, *HSS* Hospital for Special Surgery score, *WOMAC* Western Ontario McMaster University Osteoarthritis Index

^*^Statistically significant difference, *p*-value was written in italic

**Table 2:**
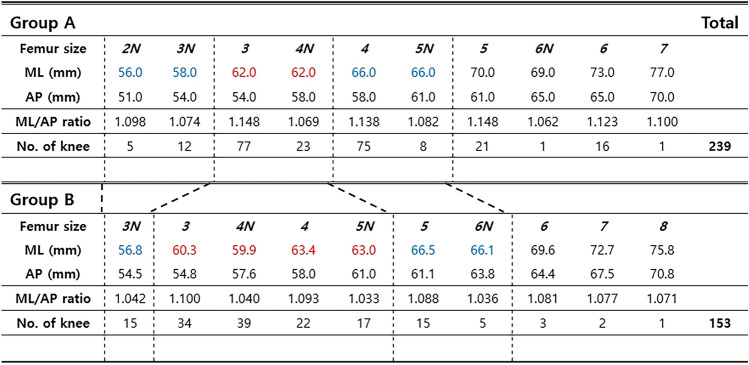
Femoral component dimension specification and the number of knees in each size

*ML* mediolateral diameter, *AP* anteroposterior diameter, *N* narrow type, *ML/AP ratio* ratio of the mediolateral to anteroposterior diameter = femoral aspect ratio, femoral component size was written in italic

**Table 3:** Femoral component size matching based on the mediolateral width

		Subgroup 1	Subgroup 2	Subgroup 3			
Group A	*Size* Knees	*2N,3N* (17)	*3,4N* (100)	*4,5N* (83)	*5,6N* (22)	*6* (16)	*7* (1)
Group B	*Size* Knees	*3N* (15)	*3,4N,4,5N* (112)	*5,6N* (20)	*6* (3)	*7* (2)	*8, 9* (1)

(): The numbers in brackets are the number of knees

**Table 4:** Femoral posterior condylar resection and trimming

	Subgroup 1			Subgroup 2			Subgroup 3		
	Group A (n = 17)	Group B (n = 15)	*P*	Group A (n = 100)	Group B (n = 112)	*P*	Group A (n = 83)	Group B (n = 20)	***P***
PLC (mm)	10.50 ± 1.39 (8.0 ~ 13.0)	8.83 ± 1.84 (5.5 ~ 11.0)	0.020*	9.28 ± 1.59 (5.0 ~ 12.5)	8.02 ± 1.97 (3.0 ~ 12.5)	< 0.001*	9.42 ± 1.79 (4.5 ~ 13.0)	7.70 ± 2.05 (4.0 ~ 11.0)	0.001*
PMC (mm)	13.29 ± 1.76 (11.0 ~ 17.0)	12.20 ± 1.89 (8.5 ~ 15.0)	*0.102*	12.20 ± 2.27 (1.0 ~ 16.0)	11.46 ± 1.91 (6.0 ~ 15.5)	< 0.001*	12.34 ± 2.06 (5.5 ~ 17.0)	10.73 ± 1.94 (6.0 ~ 14.0)	0.001*
PCT (mm)	23.79 ± 2.97 (19.0 ~ 30.0)	21.03 ± 3.61 (14.0 ~ 26.0)	0.027*	21.48 ± 3.46 (10.5 ~ 27.5)	19.47 ± 3.70 (10.0 ~ 27.5)	< 0.001*	21.75 ± 3.64 (11.0 ~ 29.0)	18.43 ± 3.55 (10.0 ~ 24.0)	0.001*
XPLC (mm)	1.00 ± 1.39 (− 1.5 ~ 3.5)	− 0.17 ± 1.84 (-3.5 ~ 2.0)	*0.153*	− 0.22 ± 1.59 (− 4.5 ~ 3.0)	− 1.48 ± 1.97 (− 5.0 ~ 3.5)	< 0.001*	− 0.08 ± 1.79 (− 5.0 ~ 3.5)	− 1.30 ± 2.05 (− 5.0 ~ 2.0)	0.033*
XPMC (mm)	3.79 ± 1.76 (1.5 ~ 7.5)	3.20 ± 1.89 (− 0.5 ~ 6.0)	*0.367*	2.70 ± 2.27 (− 8.5 ~ 6.5)	1.96 ± 1.91 (− 3.5 ~ 6.0)	< 0.001*	2.84 ± 2.06 (− 4.0 ~ 7.5)	1.73 ± 1.94 (− 3.0 ~ 5.0)	0.031*
XPCT (mm)	4.79 ± 2.97 (0.0 ~ 11.0)	3.03 ± 3.61 (− 4.0 ~ 8.0)	*0.146*	2.48 ± 3.46 (− 8.5 ~ 8.5)	0.47 ± 3.71 (− 9.0 ~ 8.5)	< 0.001*	2.75 ± 3.64 (− 8.0 ~ 10.0)	0.43 ± 3.55 (− 8.0 ~ 6.0)	0.025*

*PLC* posterolateral condyle, *PMC* posteromedial condyle, *PCT* posterior condyle total, *XPLC* extra posterolateral condylar resection except for component posterior condylar thickness, *XPMC* extra posteromedial condylar resection except for component posterior condylar thickness, *XPCT* extra total posterior condylar resection except for component bilateral posterior condylar thickness

^*^Statistically significant difference, *p*-value was written in italic

**Table 5:** Comparison of the femoral posterior condylar trimming between male and female

	Group A (n = 239)	Group B (n = 153)	*P*
Male	3.45 ± 3.57 (− 8.0 ~ 9.5) (n = 41)	− 0.05 ± 2.97 (− 7.0 ~ 4.5) (n = 21)	< 0.001*
Female	2.80 ± 3.50 (− 8.5 ~ 11.0) (n = 198)	1.69 ± 3.73 (− 8.0 ~ 9.5) (n = 132)	0.007*
*p*	*0.277*	0.043*	

*p*-value was written in italic

In Group A, narrow-type implants were used in 49 of 239 knees (20.5%); however, in Group B, 76 of 153 knees (49.7%) required narrow-type implants (Table 2) (Fig. 2).

Clinical outcomes between entire Groups A and B were compared for 85 and 38 patients who were followed up for at least 2 years after TKA. ROM were similar between the two groups as 114.06 ± 12.78 and 113.82 ± 13.97, preoperatively (p = 0.969), and 116.76 ± 17.30 and 117.89 ± 17.42, postoperatively (p = 0.803). The amount of perioperative change in ROM was statistically similar as 2.71 ± 15.82 and 4.08 ± 21.40 in group A and B (p = 0.332), respectively. HSS were similar between the two groups as 64.14 ± 12.17 and 62.47 ± 12.63, preoperatively (p = 0.273), and 87.04 ± 12.99 and 85.39 ± 10.34, postoperatively (p = 0.116). The amount of perioperative change in HSS was statistically similar as 22.89 ± 17.94 and 22.92 ± 15.28 in group A and B (p = 0.917), respectively. Preoperative WOMAC was 49.14 ± 16.08 in Group A and 57.92 ± 15.90 in Group B, which was significantly higher in Group A (p = 0.014). However, postoperative WOMAC was similar between the groups at 12.42 ± 12.64 and 12.63 ± 12.18 in Groups A and B (p = 0.854), respectively. Perioperative improvement in WOMAC was significantly larger in Group B at − 45.29 ± 15.74 than in Group A at -36.72 ± 19.06 (0.022) (Table 6).

**Table 6:** Clinical outcomes

	Group A (n = 85)	Group B (n = 38)	*P*
Preop. ROM (°)	114.06 ± 12.78 (90 ~ 135)	113.82 ± 13.97 (85 ~ 135)	*0.969*
Postop. ROM (°)	116.76 ± 17.30 (65 ~ 135)	117.89 ± 17.42 (75 ~ 135)	*0.803*
ΔROM (°)	2.71 ± 15.82 (− 35 ~ 40)	4.08 ± 21.40 (− 40 ~ 55)	*0.332*
Preop. HSS	64.14 ± 12.17 (33 ~ 90)	62.47 ± 12.63 (40 ~ 89)	*0.273*
Postop. HSS	87.04 ± 12.99 (10 ~ 100)	85.39 ± 10.34 (45 ~ 100)	*0.116*
ΔHSS	22.89 ± 17.94 (− 53 ~ 65)	22.92 ± 15.28 (− 19 ~ 53)	*0.917*
Preop. WOMAC	49.14 ± 16.08 (14 ~ 85)	57.92 ± 15.90 (26 ~ 96)	0.014*
Postop. WOMAC	12.42 ± 12.64 (0 ~ 73)	12.63 ± 12.18 (0 ~ 52)	*0.854*
ΔWOMAC	− 36.72 ± 19.06 (− 73 ~ 21)	− 45.29 ± 15.74 (− 68 ~ -6)	0.022*

*ROM* range of motion, *HSS* Hospital for Special Surgery score, *WOMAC* Western Ontario McMaster University Osteoarthritis Index, *Δ* change between the pre- and postoperative value

## Discussion

The most important finding of this study was that femoral posterior condylar resection was thicker with Asian-specific femoral implants in Korean patients. In anteriorly referenced TKA, the extent of femoral posterior condylar resection may indirectly indicate the AP diameter fit of a specific femoral component design to a patient’s distal femoral anatomy. In this study, the amount of posterior condylar resection was significantly larger in Group A than in Group B, irrespective of the implant specific posterior condylar thickness. It can be interpreted that Group A’s femoral component design more closely pursued a larger femoral aspect ratio of the Asian femora. However, significantly larger PLC, PMC, and PCT values were predictable if the femoral component in Group A was intentionally designed to have a flatter axial plane geometry than that in Group B with a similar width. Among the parameters regarding the posterior condylar trimming amount, XPLC mostly resulted in a negative value, which meant less resection amount of PLC than replacing posterolateral condylar thickness of the femoral component. XPMC resulted in a positive value, which meant trimming the extra host bone beyond the replacing posteromedial condylar thickness of the femoral component owing to the externally rotated resection of the femur in the axial plane. In particular, XPCT was the most important parameter because it neutralized the influence of individual femoral external rotation resection, and showed the actual difference between the removed bilateral posterior condyles in the host bone and replacing posterior condyles in the femoral component. Considering that the XPCT in Group B was 0.47 and 0.43 in subgroups 2 and 3, respectively, the bilateral posterior condyle of the femoral component in Group B replaced the native bilateral posterior condyle with minimal trimming. In contrast, the femoral component in Group A showed a ‘carving-in’ tendency relative to the native posterior condyle.

Several studies have been conducted on the anthropometric analysis of human knee geometry [5–15, 18]. Gender specific femoral component and asymmetric tibial component designs were developed based on anthropometric studies conducted in the 1990s. However, despite numerous studies revealing their clinical advantages, the two design modifications succeeded in better accommodating anatomical geometry but failed to prove clinical improvement compared with conventional designs [5, 24, 25]. Subsequently, the geometric differences between ethnicities have been analyzed since the 2000s, mainly regarding Caucasians, Indians, and Asians [7, 10–15]. Some studies concluded that Asian femora had a larger femoral aspect ratio than Caucasians, which led to the manufacturing of Asian-specific femoral component designs [11, 12]. To our knowledge, only two studies on this implant design have been published. Kim et al. [16] reported higher good-fitting rate of Anthem in the femoral anterior flange longitudinal area, transverse area, and distal cutting surface area compared with Attune femoral component design in their study regarding Korean population. They reported that the frequency of using narrow type option in Anthem^®^ as 18.9% and Attune^®^ as 20.2%. A narrow type implant is usually used for the knees with narrower ML dimensions on which standard type implant would likely yield ML overhang [26]. We considered that the number of femoral component size selections in Groups A and B could also reflect better accommodation of the femoral aspect ratio in the anatomically modified implant design. In our study, narrow option was utilized in 49.7% in Attune^®^ group whereas it was 20.5% in Anthem^®^ which was similar to Kim et al.’s results. The results in this study could be interpreted that the selection of narrow-type implants was less in Group A because the standard sizes of the anatomically modified implant design accommodated the Asian femoral anatomy better than the conventional design based on the ML dimension intraoperatively. On the other hand, Kim et al. [17] reported using 150 narrow type options out of 285 Anthem^®^ TKAs (52.6%). They reported that the ML dimension and mean modified aspect ration ratio was smaller in narrow-version femoral implant group than in standard group. The two studies focused mainly on the ML fitting of the femoral component, and did not consider correlation to the AP diameter. Kahlenberg et al. [27] reported the difference between posterior medial condylar resection and implant posterior condylar thickness using five different implant systems in three sawbone models. Although the study used posterior referencing resection, 0.6–2.9 mm difference was revealed in their results. Among the five implant they used, Legion^®^ (Smith & Nephew, Memphis, TN, USA) showed the largest posterior condylar trimming over the implant, which does not seem to be a mere coincidence with the Anthem^®^ from the same manufacturer. Therefore, the influence of the modified femoral aspect ratio in this specific implant on the AP diameter of the real bone and clinical results was first assessed in our study. We regarded that the results in this study was meaningful because AP diameter is closely associated with kinematics and biomechanics of the knee joint. It has been reported that a difference in flexion gap 1–3 mm might lead to changes in ROM and PROMs after TKA [28, 29]. Similarly, clinical difference in perioperative WOMAC improvement between the two groups might have been influenced by the differences in posterior condylar trimming thickness. Lower improvement in WOMAC in Group A might be implicating inferior physical functioning related to potential larger flexion gap in Group A if we consider that WOMAC reflects more physical function parameters than HSS or ROM alone. Mid-flexion or flexion instability after TKA which could be associated with flexion–extension gap imbalance often lead to pain or incapability related to stair-climbing and other knee-bending motions in activities of daily living. It is recommended that more caution should be kept during sizing, bone-resection, and gap balancing if a surgeon decide to use this modified femoral component design.

However, this study had some limitations. First, the retrospective study design limits the general application of the results. Applicability may have been improved by conducting the survey as a prospective study by controlling bilateral knees with different femoral component designs. However, we attempted to overcome this limitation by performing size-matched subgroup analyses and comparisons. Second, selecting the anterior reference point and femoral component size during the surgical procedure was highly subjective. Preferred anterior referencing point and ML sizing policy can be different between the orthopaedic surgeons. The sole surgeon in the included cases consistently referenced the starting point of the anterolateral distal femoral ridge to flush the anterior resection surface along the anterior cortex of the femoral shaft. A consistently selected underhanging femoral component size between the overhangs and underhangs relative to the distal femoral resection surface ML diameter was used. Third, the study was not powered and that the consequence of the different posterior cuts should be evaluated to see if it is associated with any further clinical meaning. Also, the clinical analysis by subgroup comparison could not be performed due to relatively small sample sizes. We tried to overcome the limitations by comparing the whole population in the two groups. Finally, clinical outcome analysis according to the preoperative diagnosis or preoperative OA grade was not performed. This was not performed because our baseline sample size was small, and seperating them would lessen the power of analysis. Further clinical analysis considering the factors need to be performed in the future.

## Conclusion

The Asian-specific femoral component design resulted in more resection and trimming of the femoral posterior condyle than the conventional design, despite the well-fitting appearance of the high femoral aspect ratio to the Asian bone resection surface, intraoperatively. Surgeons should be aware of possible excessive posterior condylar resection and formation of large flexion gap when using a femoral component design with a high aspect ratio.

## References

[1] Song SJ, Kim KI, Suh DU, Park CH (2021) Comparison of patellofemoral-specific clinical and radiographic results after total knee arthroplasty using a patellofemoral design-modified prosthesis and its predecessor. Clin Orthop Surg 13(2):175–184. 10.4055/cios20188 34094008 10.4055/cios20188 PMC8173230

[2] Hitt K, Shurman JR 2nd, Greene K, McCarthy J, Moskal J, Hoeman T et al (2003) Anthropometric measurements of the human knee: correlation to the sizing of current knee arthroplasty systems. J Bone Joint Surg Am 85-A(Suppl 4):115–122 14652402

[3] Guy SP, Farndon MA, Sidhom S, Al-Lami M, Bennett C, London NJ (2012) Gender differences in distal femoral morphology and the role of gender specific implants in total knee replacement: a prospective clinical study. Knee 19(1):28–31. 10.1016/j.knee.2010.12.005 21277212 10.1016/j.knee.2010.12.005

[4] Han SB, Yoon JR, Cheong JY, Song JH, Yoo JD, Shin YS (2022) Risk of stroke after unilateral or bilateral tka (simultaneous and staged without discharge) in 327,438 matched patients using data from the national health insurance claims for south korea. Arch Orthop Trauma Surg 142(9):2335–2348. 10.1007/s00402-021-04146-x 34462825 10.1007/s00402-021-04146-x

[5] Xie X, Lin L, Zhu B, Lu Y, Lin Z, Li Q (2014) Will gender-specific total knee arthroplasty be a better choice for women? A systematic review and meta-analysis. Eur J Orthop Surg Traumatol 24(8):1341–1349. 10.1007/s00590-013-1396-6 24370895 10.1007/s00590-013-1396-6

[6] Lim HC, Bae JH, Yoon JY, Kim SJ, Kim JG, Lee JM (2013) Gender differences of the morphology of the distal femur and proximal tibia in a Korean population. Knee 20(1):26–30. 10.1016/j.knee.2012.05.010 22721912 10.1016/j.knee.2012.05.010

[7] Ishimaru M, Hino K, Onishi Y, Iseki Y, Mashima N, Miura H (2014) A three-dimensional computed tomography study of distal femoral morphology in Japanese patients: Gender differences and component fit. Knee 21(6):1221–1224. 10.1016/j.knee.2014.09.007 25450008 10.1016/j.knee.2014.09.007

[8] Gillespie RJ, Levine A, Fitzgerald SJ, Kolaczko J, DeMaio M, Marcus RE et al (2011) Gender differences in the anatomy of the distal femur. J Bone Joint Surg Br 93(3):357–363. 10.1302/0301-620x.93b3.24708 21357958 10.1302/0301-620X.93B3.24708

[9] Yue B, Varadarajan KM, Ai S, Tang T, Rubash HE, Li G (2011) Differences of knee anthropometry between Chinese and white men and women. J Arthroplasty 26(1):124–130. 10.1016/j.arth.2009.11.020 20149574 10.1016/j.arth.2009.11.020 PMC3740371

[10] Vaidya SV, Ranawat CS, Aroojis A, Laud NS (2000) Anthropometric measurements to design total knee prostheses for the Indian population. J Arthroplasty 15(1):79–85. 10.1016/s0883-5403(00)91285-3 10654467 10.1016/s0883-5403(00)91285-3

[11] Kim TK, Phillips M, Bhandari M, Watson J, Malhotra R (2017) What differences in morphologic features of the knee exist among patients of various races? A systematic review. Clin Orthop Relat Res 475(1):170–182. 10.1007/s11999-016-5097-4 27704318 10.1007/s11999-016-5097-4 PMC5174057

[12] Chung BJ, Kang JY, Kang YG, Kim SJ, Kim TK (2015) Clinical implications of femoral anthropometrical features for total knee arthroplasty in Koreans. J Arthroplasty 30(7):1220–1227. 10.1016/j.arth.2015.02.014 25752826 10.1016/j.arth.2015.02.014

[13] Cheng FB, Ji XF, Lai Y, Feng JC, Zheng WX, Sun YF et al (2009) Three dimensional morphometry of the knee to design the total knee arthroplasty for Chinese population. Knee 16(5):341–347. 10.1016/j.knee.2008.12.019 19230678 10.1016/j.knee.2008.12.019

[14] Chaichankul C, Tanavalee A, Itiravivong P (2011) Anthropometric measurements of knee joints in Thai population: correlation to the sizing of current knee prostheses. Knee 18(1):5–10. 10.1016/j.knee.2009.12.005 20133135 10.1016/j.knee.2009.12.005

[15] Budhiparama NC, Lumban-Gaol I, Ifran NN, de Groot PCJ, Utomo DN, Nelissen R (2021) Mismatched knee implants in Indonesian and Dutch patients: a need for increasing the size. Knee Surg Sports Traumatol Arthrosc 29(2):358–369. 10.1007/s00167-020-05914-9 32162046 10.1007/s00167-020-05914-9

[16] Kim JS, Jung YS, Lee JI, Choi HG, Baek E, Yoo HJ et al (2021) Do optional implants improve the femoral fit during total knee arthroplasty in Asians? Comparison of the femoral fit between single- and dual-option implants. Knee 32:80–89. 10.1016/j.knee.2021.06.002 34454350 10.1016/j.knee.2021.06.002

[17] Kim J, Park S, Ahn JH (2022) Preoperative radiographic parameters in the case of using a narrow-version femoral implant in total knee arthroplasty. Arch Orthop Trauma Surg 142(8):2065–2074. 10.1007/s00402-021-04111-8 34405258 10.1007/s00402-021-04111-8

[18] Hosaka K, Saito S, Ishii T, Mori S, Sumino T, Tokuhashi Y (2011) Asian-specific total knee system: 5–14 year follow-up study. BMC Musculoskelet Disord 12:251. 10.1186/1471-2474-12-251 22046953 10.1186/1471-2474-12-251 PMC3231995

[19] Nishikawa M, Owaki H, Kaneshiro S, Fuji T (2014) Preoperative morphometric differences in the distal femur are based on skeletal size in Japanese patients undergoing total knee arthroplasty. Knee Surg Sports Traumatol Arthrosc 22(12):2962–2968. 10.1007/s00167-014-3253-5 25160474 10.1007/s00167-014-3253-5

[20] Kwak DS, Han S, Han CW, Han SH (2010) Resected femoral anthropometry for design of the femoral component of the total knee prosthesis in a Korean population. Anat Cell Biol 43(3):252–259. 10.5115/acb.2010.43.3.252 21212865 10.5115/acb.2010.43.3.252 PMC3015043

[21] Kamei G, Ishibashi S, Yoshioka K, Sakurai S, Inoue H, Mochizuki Y et al (2022) Evaluation of the flexion gap with a distal femoral trial component in posterior-stabilized total knee arthroplasty. Knee Surg Relat Res 34(1):10. 10.1186/s43019-022-00142-6 35272708 10.1186/s43019-022-00142-6 PMC8908638

[22] Bae SC, Lee HS, Yun HR, Kim TH, Yoo DH, Kim SY (2001) Cross-cultural adaptation and validation of Korean western ontario and mcmaster universities (womac) and lequesne osteoarthritis indices for clinical research. Osteoarthr Cartil 9(8):746–750. 10.1053/joca.2001.0471 10.1053/joca.2001.0471 11795994

[23] Insall JN, Ranawat CS, Aglietti P, Shine J (1976) A comparison of four models of total knee-replacement prostheses. J Bone Joint Surg Am 58(6):754–765 956219

[24] Dai Y, Scuderi GR, Penninger C, Bischoff JE, Rosenberg A (2014) Increased shape and size offerings of femoral components improve fit during total knee arthroplasty. Knee Surg Sports Traumatol Arthrosc 22(12):2931–2940. 10.1007/s00167-014-3163-6 25026932 10.1007/s00167-014-3163-6 PMC4237918

[25] Xie X, Zhong Y, Lin L, Li Q (2015) No clinical benefit of gender-specific total knee arthroplasty: a systematic review and meta-analysis of 6 randomized controlled trials. Acta Orthop 86(2):274–275. 10.3109/17453674.2015.1022107 25708427 10.3109/17453674.2015.1022107 PMC4404785

[26] Mahoney OM, Kinsey T (2010) Overhang of the femoral component in total knee arthroplasty: risk factors and clinical consequences. J Bone Joint Surg Am 92(5):1115–1121. 10.2106/jbjs.H.00434 20439656 10.2106/JBJS.H.00434

[27] Kahlenberg CA, Elmasry S, Mayman DJ, Cross MB, Wright TM, Westrich GH et al (2019) Posterior condylar bone resection and femoral implant thickness vary by up to 3 mm across implant systems: Implications for flexion gap balancing. Knee Surg Sports Traumatol Arthrosc 27(7):2140–2144. 10.1007/s00167-019-05422-5 30820600 10.1007/s00167-019-05422-5

[28] Ismailidis P, Kuster MS, Jost B, Giesinger K, Behrend H (2017) Clinical outcome of increased flexion gap after total knee arthroplasty. Can controlled gap imbalance improve knee flexion? Knee Surg Sports Traumatol Arthrosc 25(6):1705–1711. 10.1007/s00167-016-4009-1 26846656 10.1007/s00167-016-4009-1

[29] Christen B, Heesterbeek P, Wymenga A, Wehrli U (2007) Posterior cruciate ligament balancing in total knee replacement: the quantitative relationship between tightness of the flexion gap and tibial translation. J Bone Joint Surg Br 89(8):1046–1050. 10.1302/0301-620X.89B8.18976 17785743 10.1302/0301-620X.89B8.18976

